# Antiphospholipid antibody profiles in lupus nephritis with glomerular microthrombosis: a prospective study of 124 cases

**DOI:** 10.1186/ar2736

**Published:** 2009-06-22

**Authors:** Hui Zheng, Yi Chen, Wen Ao, Yan Shen, Xiao-wei Chen, Min Dai, Xiao-dong Wang, Yu-cheng Yan, Cheng-de Yang

**Affiliations:** 1 Department of Rheumatology, Renji Hospital, Shanghai Jiaotong University School of Medicine, 145 Shan Dong Zhong Road, Shanghai, 200001, PR China; 2 Department of Nephrology, Renji Hospital, Shanghai Jiaotong University School of Medicine, 145 Shan Dong Zhong Road, Shanghai, 200001, PR China

## Abstract

**Introduction:**

Glomerular microthrombosis (GMT) is a common vascular change in patients with lupus nephritis (LN). The mechanism underlying GMT is largely unknown. Although several studies have reported the association of antiphospholipid antibodies (aPL) with GMT, the relation between GMT and aPL remains controversial. Previous studies have demonstrated that some aPL could bind to several hemostatic and fibrinolytic proteases that share homologous enzymatic domains. Of the protease-reactive aPL, some can inhibit the anticoagulant activity of activated protein C and the fibrinolytic function of plasmin, and hinder the antithrombin inactivation of thrombin. The purpose of this study was to investigate the prevalence of GMT in LN patients and examine the relation between the aPL profiles (including some protease-reactive aPL) and GMT.

**Methods:**

Renal biopsy specimens were examined for the presence of glomerular microthrombi. Plasma samples from 25 LN patients with GMT (LN-GMT group) and 99 LN patients without GMT (LN-non-GMT group) were tested for lupus anticoagulant and antibodies against cardiolipin, β2 glycoprotein I, plasmin, thrombin, tissue plasminogen activator, and annexin II.

**Results:**

The prevalence of GMT in LN patients was 20.2%. Compared with the LN-non-GMT group, the LN-GMT group had an elevated systemic lupus erythematosus disease activity index; elevated renal tissue injury activity and chronicity indices; elevated serum creatinine, blood urea nitrogen, and proteinuria levels; a lower serum C3 level and much intense glomerular C3, C1q staining; and a higher frequency of hypertension (*P *< 0.05 for all). Additionally, the detection rate of lupus anticoagulant, immunoglobulin G (IgG) anti-β2 glycoprotein I and anti-thrombin antibodies were higher in the LN-GMT group than in the LN-non-GMT group (*P *< 0.05 for all). No statistical differences were found in the detection rates of IgG anti-cardiolipin, plasmin, tissue plasminogen activator, or annexin II antibodies (*P *> 0.05 for all). No detectable difference in IgM autoantibodies to the above antigens was observed between the two groups.

**Conclusions:**

GMT occurs in approximately 20.2% of LN patients. Patients with GMT have severer renal tissue injuries and poorer renal functions than patients without GMT. The lupus anticoagulant and antibodies against β2 glycoprotein I and thrombin may play a role in GMT.

## Introduction

Systemic lupus erythematosus (SLE) is a multisystem autoimmune disease. Approximately 40 to 85% of SLE patients develop renal involvement, lupus nephritis (LN), which is characterized by proteinuria, hematuria, and cylindruria, and even renal failure in some cases during the course of the disease [1-3]. Glomerular microthrombosis (GMT) is seen in approximately 30 to 33% of patients with LN and it is especially seen in those with severe diffuse proliferative glomerulonephritis [4,5]. Previous studies have indicated that LN patients with GMT have more severe renal tissue injuries, poorer responses to routine treatments, and worse renal outcomes than patients without GMT [5-12]. Thus, apart for the fact that the immune complex could directly elicit glomerular injuries, GMT may be another important cause of renal injury and dysfunction in a subset of LN patients.

Antiphospholipid antibodies (aPL) are a heterogeneous group of antibodies directed against negatively-charged phospholipids, phospholipid-binding proteins, and phospholipid-protein complexes. The laboratory criteria of update criteria for definite antiphospholipid syndrome (APS) includes the lupus anticoagulant (LAC), the anticardiolipin antibody (aCL), and the anti-β2 glycoprotein I (β2GPI) antibody [13]. GMT has been associated with aPL in LN patients in some studies [4,6,7,9-12,14-18], but not in others [5,8,19-21]. Recent studies of seven monoclonal immunoglobulin (Ig) G aCLs from two APS patients demonstrated that five aCLs reacted with several hemostatic and fibrinolytic proteases that share homologous enzymatic domains. Autoantibodies to these proteases, including thrombin, plasmin, tissue plasminogen activator (t-PA), prothrombin, protein C, protein S, annexin II (A2), annexin V, and coagulation factor X, were found in APS patients [22-28]. Importantly, our previous studies have demonstrated that some protease-reactive monoclonal IgG aCL can interfere with the inactivation of thrombin by antithrombin and decrease the function of plasmin and activated protein C [22,23,29]. In addition, aPL may bind to A2 and inhibit A2-dependent plasmin generation [30]. Therefore, aPL may promote various thrombotic events by interacting with these hemostatic and fibrinolytic proteases [31]. It is of interest to investigate whether some protease-reactive aPL are present in LN patients with GMT. In order to address this, we carried out a prospective study of 124 LN patients undergoing renal biopsy to further investigate the prevalence of GMT and examine the significance of aPL in LN patients with GMT.

## Materials and methods

### Patients

The study comprised 124 consecutive patients with LN who had been referred to the Renji Hospital at the Shanghai Jiaotong University School of Medicine for renal biopsy between September 2007 and October 2008. All patients fulfilled the American College of Rheumatology classification criteria for the diagnosis of SLE [32]. In addition, all patients had clinical evidence of LN, which was further proven by pathologic examination of renal biopsy specimens.

Plasma samples were collected on the day of renal biopsy. The following demographic, clinical, and serologic data were collected at the time of the renal biopsy: sex; age; duration of SLE and LN; history of symptomatic thrombosis; levels of blood urea nitrogen, serum creatinine, serum C3, C4, C1q and proteinuria; prevalence of systemic hypertension; and presence or absence of antinuclear antibodies (ANA), anti-Sm, anti-ribonucleoprotein (anti-RNP), anti-double-stranded DNA (anti-dsDNA), anti-histone, and anti-nucleosome antibodies. The systemic lupus erythematosus disease activity index (SLEDAI) was used to estimate global disease activity.

In addition, another 100 healthy adults were randomly recruited to serve as normal controls. All patients were carefully examined if they have other potential causes for GMT, such as systemic sclerosis, thrombotic thrombocytopenic purpura/hemolytic uremic syndrome, malignant hypertension, diabetic nephropathy, postpartum renal failure, preeclampsia, HIV infection, or cyclosporine therapy [6,8].

The patients were informed of the purpose of the study and gave their informed consent. The institutional review board of Shanghai Jiaotong University approved this study.

### Renal histology

All patients underwent ultrasound-guided renal needle biopsy. The renal tissues obtained by biopsy were fixed in 10% neutral buffered formalin, gradually dehydrated, and embedded in paraffin. Paraffin sections were stained with H&E, periodic acid-Schiff, Masson's trichrome, and periodic acid-silver methenamine. Small portions of fresh renal tissue were snap frozen and 4 μm cryostat-cut sections were incubated with fluorescein isothiocyanate conjugate (FITC)-conjugated rabbit antisera against human IgG, IgA, IgM, C1q, or C3 (Dako, Glostrup, Denmark) and were examined by direct immunofluorescence [8]. Biopsy specimens were classified using the International Society of Nephrology/Renal Pathology Society (ISN/RPS) 2003 classification of LN [33]. In addition, particular attention was paid to GMT. Thrombosis was considered to be present when thrombi with fibrin-consistent staining properties were clearly seen by light microscopy occluding the glomerular capillary lumens. In order to confirm the presence of fibrin GMT, cryostat sections were also incubated with FITC-conjugated rabbit antiserum against human fibrinogen (Dako, Glostrup, Denmark). When necessary, laser confocal microscopy was used to further determine whether the microthrombi were within the glomerular capillary lumens or not. The patients were divided into two groups (LN-GMT group and LN-non-GMT group) based on the presence or absence of GMT.

### Activity and chronicity indices of renal tissue injury

Renal tissue injury was evaluated using activity and chronicity indices as previously reported by Austin and colleagues [34]. The activity index was the sum of the scores (on a scale of 1 to 3) for endocapillary proliferation, karyorrhexis, fibrinoid necrosis (with the score for fibrinoid necrosis multiplied by 2), cellular crescents (with the score multiplied by 2), hyaline deposits, leukocyte exudation, and interstitial inflammation. The score on the chronicity index was the sum of the scores (on a scale of 1 to 3) for glomerular sclerosis, fibrous crescents, tubular atrophy, and interstitial fibrosis.

### Immune complex deposits

The intensity of glomerular immunofluorescence staining for IgG, IgM, IgA, C3, and C1q was semiquantitatively scored on a scale of 0 to 3, where 0 = no glomerular staining, 1 = mild glomerular staining, 2 = moderate glomerular staining, and 3 = intense glomerular staining [19].

### LAC assay

LAC was detected using a LAC Screen/LAC Confirm Kit (Instrumentation Laboratory Company, Lexington, MA, USA) on the IL coagulation system (Instrumentation Laboratory Company, Lexington, MA, USA) to determine the dilute Russell's viper venom time according to the manufacturer's instructions. The results of LAC are expressed as normalized LAC ratios, and ratios more than 1.2 were considered positive.

### aCL assay

The presence of IgG and IgM aCL were detected using quantitative ELISA kits (EUROIMMUN Medizinische Labordiagnostika AG, Lübeck, Germany) according to the manufacturer's instructions. The levels of aCL were expressed as standard units for either G phospholipid (GPL) units/ml or M phospholipid (MPL) units/ml and values more than 12 GPL units/ml or more than 12 MPL units/ml were considered positive.

### Anti-β2GPI antibodies

IgG and IgM anti-β2GPI antibodies were measured with ELISA kits (EUROIMMUN Medizinische Labordiagnostika AG, Lübeck, Germany). The levels of anti-β2GPI antibodies were expressed in relative units and values of more than 20 RU/ml were considered positive for either the IgG or IgM isotype.

### Assay for anti-plasmin, thrombin, t-PA, and A2 antibodies

For anti-plasmin, anti-thrombin, and anti-t-PA antibodies detection, high-binding ELISA plates (Costar, Cambridge, MA, USA) were coated with 5 μg/ml of human plasmin or α-thrombin (Haematologic Technologies, Essex Junction, VT, USA) or 10 μg/ml human t-PA (Merck KGaA, Darmstadt, Germany) in 0.01 M PBS, pH 7.4. Following an overnight incubation at 4°C, the plates were blocked with PBS containing 0.3% gelatin and incubated for two hours at 37°C. Plasma samples were diluted with PBS containing 0.1% gelatin, plated in duplicate, and incubated for one hour at 37°C. After washing with PBS containing 0.1% Tween-20, the bound human IgG was detected with affinity-isolated, antigen-specific, horseradish peroxidase-conjugated goat anti-human IgG (Fc specific; Sigma-Aldrich, St. Louis, MO, USA). After an additional incubation for one hour at 37°C, 100 μl of the tetramethylbenzidine/hydrogen peroxidase substrate solution (Kirkegard & Perry Labs, Gaithersburg, MD, USA) was added and the reaction terminated with 50 μl of 0.5 M sulfuric acid. The results were read at a wavelength of 450 nm in a microplate reader (Bio-Rad Laboratories, Hercules, CA, USA). The ELISA for the detection of anti-A2 antibodies was similar except for the following modifications. The wells were coated with 10 μg/ml (in PBS) human A2 generated in our laboratory (Ao W et al, unpublished observations). The bound human IgG or IgM against A2 were all detected with affinity-isolated, antigen-specific, horseradish peroxidase-labeled goat anti-human IgG or IgM (Fc specific; Sigma-Aldrich, St. Louis, MO, USA). For each of the above antibodies, the mean absorbance plus three times the standard deviation (SD) of the normal controls was used as the cutoff for determining positivity.

### Statistical analysis

Categorical data between different groups were compared by chi-squared test or Fisher's exact test when required. For continuous variables, the comparisons were carried out using the student's t-test for two independent samples or the Mann-Whitney U test for non-normal data. *P *values less than 0.05 were considered statistically significant.

## Results

### Demographic, clinical, and laboratory characteristics of the LN patients

This study examined 124 LN patients (105 women and 19 men) with a mean age (± SD) of 33 ± 14 years. There were 25 patients in the LN-GMT group (22 women and 3 men) and 99 patients in the LN-non-GMT group (83 women and 16 men). The mean age (± SD) of the two groups was 35 ± 14 and 33 ± 14 years, respectively. Among the 100 normal controls, 85 were women and 15 were men. The mean age (± SD) of the control group was 35 ± 10 years. No significant difference was seen among the three groups in terms of age or gender (*P *> 0.05 for all).

SLEDAI, blood urea nitrogen, serum creatinine, and proteinuria levels were all significantly greater in the LN-GMT group than in the LN-non-GMT group (*P *< 0.05 for all). In addition, patients in the LN-GMT group also had a higher frequency of systemic hypertension (*P *< 0.05). The serum C3 level was significantly lower in the LN-GMT group than in the LN-non-GMT group (*P *< 0.05). However, the duration of SLE or LN, the level of serum C4 or C1q, as well as the antecedent history of thrombosis, was not statistically different between the two groups (*P *> 0.05 for all). Except for a lower frequency of anti-Sm antibodies in the LN-GMT group (*P *< 0.05), we failed to find any association between GMT and ANA, anti-ribonucleoprotein, anti-dsDNA, anti-histone, or anti-nucleosome antibodies (*P *> 0.05 for all; Table 1). None of the 124 patients had other potential causes for GMT as described previously.

**Table 1:** Demographic, clinical, and laboratory characteristics of the LN patients*

	LN-GMT (n = 25)	LN-non-GMT (n = 99)	*P *value
Sex (male/female)	3/22	16/83	0.837
Age (years)	35 ± 14	33 ± 14	0.485
SLE duration (months)	48 (8 to 126)	19 (4 to 69.5)	0.096
LN duration (months)	19 (1 to 78)	4.5 (1–24)	0.101
SLEDAI	17 ± 6	12 ± 6	0.002
Proteinuria (g/24 hours)	3.24 (2.22 to 4.66)	2.01 (1.19 to 3.66)	0.005
Serum creatinine (μmol/L)	99.7(67.85 to 118.5)	59.6(49.08 to 82.75)	<0.001
Blood urea nitrogen (mmol/L)	11.3 (6.05 to 15)	6.4 (4.9 to 10)	0.007
Serum C3 (g/L)	0.44 ± 0.23	0.62 ± 0.26	0.003
Serum C4 (g/L)	0.09 (0.06 to 0.17)	0.10 (0.05 to 0.17)	0.717
Serum C1q (g/L)	0.38 (0.34 to 0.43)	0.33 (0.29 to 0.40)	0.092
ANA (positive/negative)^†^	20/3	82/8	0.837
Anti-Sm (positive/negative)	3/20	32/58	0.037
Anti-RNP (positive/negative)	4/19	34/56	0.065
Anti-dsDNA (positive/negative)^‡^	20/5	67/29	0.312
Anti-nucleosome(positive/negative)^¶^	10/6	21/25	0.246
Anti-histone (positive/negative)^§^	10/5	30/19	0.703

*Except where indicated otherwise, values are expressed as mean ± standard deviation or median (25th–75th percentile) as appropriate.

^† ^In two patients in the LN-GMT group and nine patients in the LN-non-GMT group, ANA, anti-RNP, and anti-Sm antibodies were not detected.

^‡ ^In three patients in the LN-non-GMT group, anti-dsDNA antibodies were not detected.

^¶ ^In nine patients in the LN-GMT group and fifty three patients in the LN-non-GMT group, anti-nucleosome antibodies were not detected.

^§ ^In 10 patients in the LN-GMT group and 50 patients in the LN-non-GMT group anti-histone antibodies were not detected.

ANA = antinuclear antibodies; anti-dsDNA = anti-double-stranded DNA antibody; anti-RNP = anti-ribonucleoprotein antibody; GMT = glomerular microthrombosis; LN = lupus nephritis; SLE = systemic lupus erythematosus; SLEDAI = systemic lupus erythematosus disease activity index.

### Renal biopsy findings

**Figure 1:**
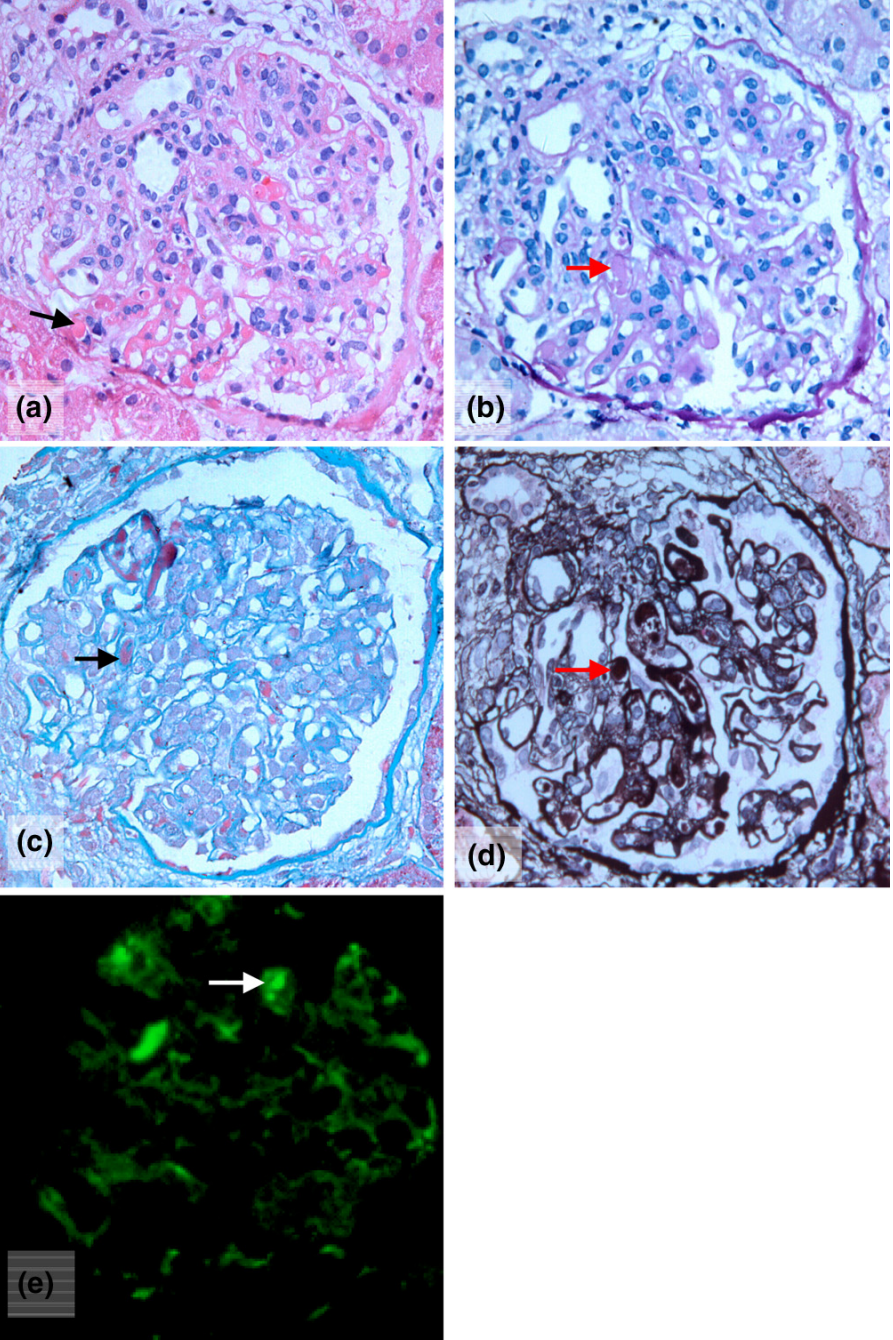
Glomerular microthrombi in the renal biopsy specimens of a patient. **(a) **Fibrin microthrombi stained red (black arrow; hematoxylin and eosin stained). **(b) **Fibrin microthrombi stained red (red arrow; Masson's trichrome stained). **(c) **Fibrin microthrombi stained purple (black arrow; periodic acid-Schiff stained). **(d) **Fibrin microthrombi stained dark brown (red arrow; periodic acid-silver methenamine stained). **(e) **Microthrombi containing fibrin/fibrinogen within the glomerular capillary lumen (white arrow; direct immunofluorescent staining of fibrinogen). Magnification: ×400.

The presence of GMT was detected in 25 of the 124 patients both by light microscopy and immunofluorescence microscopy (Figure 1). The distribution of the ISN/RPS classification of the 124 patients was as follows: 3 were class I, 2 were class II, 15 were class III, 39 were class IV, 28 were class V, 21 were class (III + V), 16 were class (IV + V), and no patients were class VI (Table 2). Class IV LN was the most frequently observed form in the LN-GMT group (64%), while class V had a slightly higher frequency (28.3%) than any other class in the LN-non-GMT group. Among the patients with class IV LN, 41% developed GMT. No GMT was detected in patients with class I, II, or III LN.

**Table 2:** Comparison between histologic parameters of LN-GMT and LN-non-GMT groups*

	LN-GMT (n = 25)	LN-non-GMT (n = 99)	*P *value
ISN/RPS classification			<0.001^†^
I	0 (0)	3 (3.0)	
II	0 (0)	2 (2.0)	
III	0 (0)	15 (15.2)	
IV	16 (64.0)	23 (23.2)	
V	0 (0)	28 (28.3)	
VI	0 (0)	0 (0)	
III + V	2 (8.0)	19 (19.2)	
IV + V	7 (28.0)	9 (9.1)	
Activity index	8 (6 to 9.5)	3 (2 to 5)	<0.001
Chronicity index	3 (2 to 4)	2 (1 to 3)	0.004

* Except where indicated otherwise, values are the number (%) of patients or median (25th–75th percentile) as appropriate.

^† ^*P *value for the difference in the ISN/RPS classification distribution between the two groups.

GMT = glomerular microthrombosis; ISN = International Society of Nephrology; LN = lupus nephritis; RPS = Renal Pathology Society.

When compared with the LN-non-GMT group, the LN-GMT group was more likely to be associated with class IV LN (*P *< 0.05). The activity and chronicity indices were also significantly higher in the LN-GMT group than in the LN-non-GMT group (*P *< 0.05 for all), with median (25th–75th percentile) activity index values of 8 (6 to 9.5) and 3 (2 to 5), respectively, and median (25th–75th percentile) chronicity index values of 3 (2 to 4) and 2 (1 to 3), respectively (Table 2). A significant relation was found between the presence of GMT and the intensity of glomerular IgM, C3, or C1q staining (*P *< 0.05 for all; Table 3).

**Table 3:** Relation between immune complex deposits and the presence of GMT*

	LN-GMT (n = 25)	LN-non-GMT (n = 99)	P value
IgG	2 (1 to 2)	2 (1 to 2)	0.967
IgA	1 (0 to 2)	1 (1 to 1)	0.253
IgM	1 (1 to 2)	0 (0 to 1)	0.001
C3	2 (1 to 2)	1 (0 to 2)	0.005
C1q	2 (1 to 2)	1 (1 to 2)	0.003

*Except where indicated otherwise, values are expressed as median (25th to 75th percentile).

GMT = glomerular microthrombosis; Ig = immunoglobulin; LN = lupus nephritis.

### aPL profiles in LN patients with or without GMT

**Figure 2:**
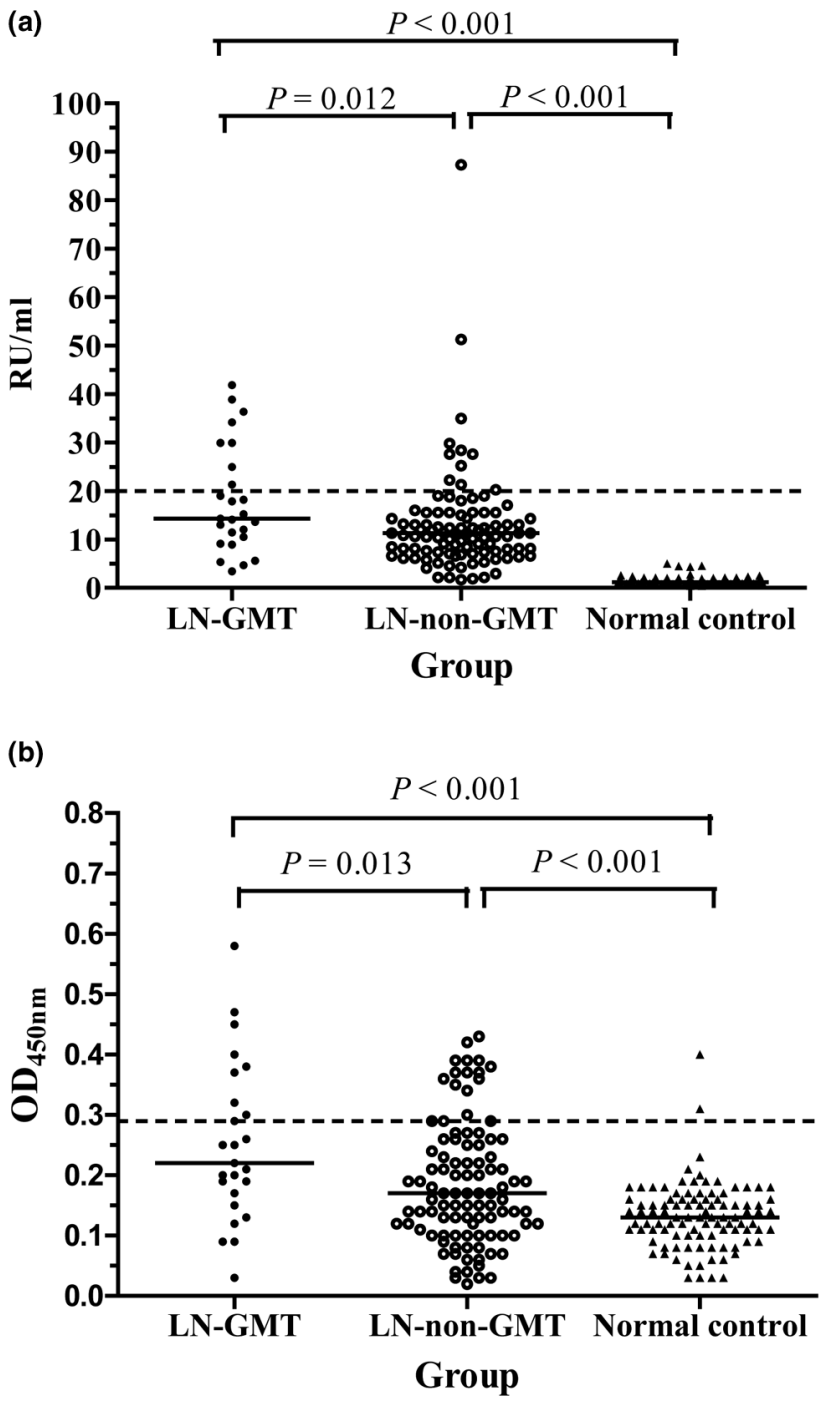
Presence of IgG anti-β2GPI and anti-thrombin antibodies in 124 LN patients. **(a) **Plasma samples from 25 patients with lupus nephritis and glomerular microthrombosis (LN-GMT), 99 patients without GMT (LN-non-GMT), and 100 normal controls were analyzed for IgG anti-β2 glycoprotein I (β2GPI) antibodies at a dilution of 1:201. Horizontal bars indicate the median values; the dashed line represents the cutoff value (20 RU/ml). **(b) **Plasma samples from LN-GMT group, LN-non-GMT group, and 100 normal controls were analyzed for IgG anti-thrombin antibodies at a dilution of 1:100. Horizontal bars indicate the median OD for each group; the dashed line represents the cutoff, which is mean OD+ three standard deviations of the 100 normal controls. Results are representative of two experiments.

LAC was detected in 7 (28.0%) of the 25 LN patients with GMT and in 8 (8.1%) of the 99 patients without GMT. IgG anti-β2GPI antibodies were detected in 32.0% of the LN-GMT group and 11.1% of the LN-non-GMT group (Figure 2a). Nine patients (36.0%) in the LN-GMT group and 17 patients (17.2%) in the LN-non-GMT group had significant levels of IgG anti-thrombin antibodies (Figure 2b). GMT was strongly associated with LAC, IgG anti-β2GPI, and anti-thrombin antibodies (*P *< 0.05 for all; Table 4; Figure 2). The detection rate of IgG aCL, anti-plasmin, anti-t-PA, or anti-A2 antibodies in the LN-GMT group was not statistically different from the LN-non-GMT group (*P *> 0.05 for all; Table 4). IgM aCL, anti-β2GPI, and anti-A2 antibodies were also detected and no significant differences were observed in the detection rate of any IgM antibody between the two groups (*P *> 0.05 for all; data not shown).

**Table 4:** Association between GMT and aPL profiles*

	LN-GMT (n = 25)	LN-non-GMT (n = 99)	*P *value
LAC	7 (28.0)	8 (8.1)	0.017
aCL	11 (44.0)	27 (27.3)	0.105
Anti-β2GPI antibodies	8 (32.0)	11 (11.1)	0.023
Anti-thrombin antibodies	9 (36.0)	17 (17.2)	0.039
Anti-plasmin antibodies	8 (32.0)	16 (16.2)	0.132
Anti-t-PA antibodies	3 (12.0)	9 (9.1)	0.951
Anti-A2 antibodies	7 (28.0)	14 (14.4)	0.176

*Except where indicated otherwise, values are the number (%) of patients.

A2 = annexin II; aCL = anticardiolipin antibody; aPL = antiphospholipid antibodies; β2GPI = β2 glycoprotein I; GMT = glomerular microthrombosis; LAC = lupus anticoagulant; LN = lupus nephritis; t-PA = tissue plasminogen activator.

## Discussion

GMT is a common vascular change in patients with LN, especially in those with severe diffuse proliferative glomerulonephritis. This prospective study of 124 consecutive patients undergoing renal biopsy demonstrates that GMT occurred in approximately 20.2% of the LN patients. This prevalence is lower than has been reported by other groups (approximately 30 to 33%) [4,5], which may be due to differences in patient selection. Consistent with previous studies [4,5,16], we also found that GMT was associated with class IV LN (i.e., diffuse proliferative glomerulonephritis). In our study, 41% of the patients with class IV LN developed GMT.

Microthrombi could mechanically obstruct glomerular capillaries, diminishing the blood supply to glomeruli and renal tubules, thereby causing chronic hypoxic/ischemic injuries to the affected glomeruli and tubules. This would, in turn, decrease the glomerular filtration rate leading to the loss of nephrons and impair renal function. Clinical studies have indicated that LN patients with GMT have more severe renal tissue injuries, poorer responses to general treatment, and worse renal outcomes than patients without GMT [4-12]. Consistent with these findings, we demonstrate in the present study that LN patients with GMT have higher activity and chronicity indices than those without GMT. The SLEDAI score and the levels of blood urea nitrogen, serum creatinine, and proteinuria, as well as the frequency of systemic hypertension, were all significantly greater in patients with GMT. Taken together, GMT may be an important cause of renal injury and renal dysfunction in a subset of patients with LN. Nevertheless, the mechanisms underlying GMT in patients with LN remain obscure.

aPL, including LAC, aCL, and anti-β2GPI antibodies, are considered to be of pathogenic significance in thrombosis in APS and SLE patients, which makes them the most frequently examined factors in the investigation of the pathogenesis of GMT in LN. GMT has been found to be associated with LAC and/or aCL in LN patients in some studies [4,6,7,9-12,14-18], but not in others [5,8,19-21]. Kant and colleagues [4] examined 105 kidney biopsy specimens from LN patients and found that GMT was detected in 34 cases, among which 7 were LAC positive. A strong association was observed between the detection of LAC and GMT. Bhandari and colleagues [7] reported that the frequency of aCL was 60% in LN patients with GMT, which was statistically higher than in patients without GMT. They considered aCL as a strong predictor of GMT in LN. However, Miranda and colleagues [5] investigated the frequency and distribution of GMT in 108 renal biopsies from Mexican lupus patients and found that GMT was not associated with aCL. Antiphospholipid syndrome nephropathy (APSN), the intrarenal vascular involvement attributable to primary or secondary APS, has recently aroused increasing research attention [6,8,35-43]. According to previously published reports[8,36], APSN includes acute lesion, that is, thrombotic microangiopathy, and chronic lesions, that is, fibrous intimal hyperplasia, organized thrombi with or without recanalization, fibrous arterial and arteriolar occlusion, and focal cortical atrophy. APSN occurs in SLE and is independent of LN. In a retrospective study carried out on 150 cases, Cheunsuchon and colleagues [43] demonstrated that the prevalence of APSN in Thai SLE patients who underwent renal biopsies was 34%. In this widely investigated entity, GMT is defined as an acute event. As chronic APSN often developed from acute APSN [6], and titers of aPL may vary in different stages of disease [13], we only analyzed the association between acute APSN and aPL in this study. A group of French investigators evaluated the incidence of APSN in 114 patients with LN and found that 55% of the patients with acute APSN were LAC positive. Acute APSN was associated with LAC but not with aCL [8]. In our study, we also found an association between LAC and GMT, but failed to find any association between IgG or IgM aCL and GMT. This probably indicates that GMT may be associated with aPL that recognize antigens such as β2GPI and some hemostatic and fibrinolystic proteases instead of cardiolipin.

β2GPI may act as a cofactor of aPL in inhibiting phospholipid-dependent coagulation. IgG and IgM anti-β2GPI antibodies assays have been added in the revised criteria (Sydney 2006 International Classification criteria for APS) [13]. Previous studies have found that the prevalence of anti-β2GPI antibodies in LN patients was much higher than in non-LN patients [44,45]. Our study is the first to investigate the association between anti-β2GPI antibodies and GMT in LN. We found that the titers and the frequency of IgG anti-β2GPI antibodies in patients with GMT were markedly higher than in patients without GMT, which indicates that anti-β2GPI antibodies may have a role in GMT.

Increasing evidence revealed by *in vivo *and *in vitro *studies have indicated that aPL may influence the dynamic equilibrium between hemostasis and fibrinolysis by cross-reacting with some hemostatic and fibrinolytic proteases (e.g., plasmin, thrombin, t-PA, protein C, protein S, A2, annexin V, and coagulation factor X) thereby facilitating various kinds of thrombotic events [22-28]. Our previous studies have demonstrated an association between some protease-reactive aPL and APS, thrombotic events, and pulmonary arterial hypertension in SLE patients [46,47]. To our knowledge, however, there has been no reports examining the relation between these antibodies and GMT in patients with LN. We found that anti-thrombin antibodies can be detected in the plasma of LN patients. The positive rate of anti-thrombin antibodies in patients with GMT was 36.0%, which was significantly higher than in patients without GMT. The titers of anti-thrombin antibodies were also higher in patients with GMT. Hwang and colleagues suggested that some anti-thrombin antibodies may bind to thrombin and interfere with the thrombin-antithrombin interaction and thus reduce the antithrombin inactivation of thrombin, and as a result contribute to thrombosis [22]. Our previous study has found that one of the cardiolipin-induced autoantibodies, CL15, bound to plasmin and was able to reduce the plasmin-mediated lysis of fibrin clots *in vitro *[23]. In this study, we found that the detection rate of IgG anti-plasmin antibodies in patients with GMT was slightly, but not statistically, higher than in patients without GMT, which is probably because anti-plasmin antibodies are not the major antibodies contributing to the development of GMT in LN. However, this presumption should be made with caution, as previous studies have demonstrated a positive association of anti-plasmin antibodies with thrombosis in both APS and SLE [46,47]. Therefore, it will be necessary to further investigate the role of anti-plasmin antibodies in LN with GMT. Additionally, no association between anti-t-PA antibodies and GMT was observed. However, this may be due to a conformational change of t-PA under different conditions [24]. The detection of anti-A2 antibodies in the sera of APS patients also suggests an important role of A2 in hemostasis and fibrinolysis [30,48]. For the first time, we evaluated the levels of IgG and IgM anti-A2 antibodies in LN patients and found that the anti-A2 antibody prevalence by IgG or IgM isotype was 16.9% and 10.5%, respectively. However, no association between IgG or IgM anti-A2 antibody and GMT in LN was observed. This may be due to the fact that aPL bind to A2 indirectly, and require assistance from cofactors such as β2GPI [49].

Many studies have shown that complement activation may play an important role in thrombotic events. aPL may activate the complement pathway, generating split products that lead to fetal loss and thrombosis [31,50,51]. Pierangeli and colleagues [52] demonstrated that C3- and C5-deficient mice were resistant to aPL-induced thrombosis. Nangaku and colleagues [53] found that temporarily inhibiting C5b-9 (the membrane attack complex) could prevent renal thrombotic microangiopathy. We also demonstrated an association between GMT and complement activation. Patients with GMT had a lower serum C3 level and much intense glomerular C3, C1q staining than those without GMT. These findings imply that complement activation, induced by or coordinated with aPL, may be essential to GMT.

## Conclusions

The prospective study carried out on 124 patients undergoing renal biopsy demonstrates that GMT occurs in approximately 20.2% of LN patients and that LAC and autoantibodies against β2GPI and thrombin play a role in GMT in LN. However, the mechanisms by which these antibodies induce GMT remain largely unknown. aPL may activate the complement pathway, generating split products that lead to thrombosis. Taking into account the important role of GMT in LN prognosis, whether those patients with renal biopsy-proven GMT should be treated with anticoagulants in the absence of other thrombotic processes has become a problem that urgently needs to be solved. It will be important in the future to carry out pertinent long-term prospective studies in a broad spectrum of the representative population and to test this hypothesis in animal models.

## Abbreviations

A2: annexin II; aCL: anticardiolipin antibody; ANA: antinuclear antibodies; anti-dsDNA: anti-double-stranded DNA antibody; anti-RNP: anti-ribonucleoprotein antibody; aPL: antiphospholipid antibodies; APS: antiphospholipid syndrome; APSN: antiphospholipid syndrome nephropathy; β2GPI: β2 glycoprotein I; ELISA: enzyme-linked immunosorbent assay; FITC: fluorescein isothiocyanate conjugate; GMT: glomerular microthrombosis; GPL: G phospholipid; H&E: hematoxylin and eosin; Ig: immunoglobulin; ISN: International Society of Nephrology; LAC: lupus anticoagulant; LN: lupus nephritis; MPL: M phospholipid; PBS: phosphate-buffered saline; RPS: Renal Pathology Society; SD: standard deviation; SLE: systemic lupus erythematosus; SLEDAI: systemic lupus erythematosus disease activity index; t-PA: tissue plasminogen activator.

## Competing interests

The authors declare that they have no competing interests.

## Authors' contributions

HZ and YC performed most of the experiments and prepared the manuscript. AW performed the majority of the A2 generation and participated in the statistical analysis. YS and X-WC worked on the clinical data presentation. MD and X-DW performed the majority of the renal tissue preparation. Y-CY performed the light microscopy and immunofluorescence analysis. C-DY was responsible for the main experimental design, data interpretation, and for finalizing the manuscript. All authors read and approved the final manuscript.
